# Effect of Cellulases and Xylanases on Refining Process and Kraft Pulp Properties

**DOI:** 10.1371/journal.pone.0161575

**Published:** 2016-08-24

**Authors:** Kamila Przybysz Buzała, Piotr Przybysz, Halina Kalinowska, Małgorzata Derkowska

**Affiliations:** 1 Institute of Papermaking and Printing Technology, Lodz University of Technology, Wolczanska str. 223, 90-924, Lodz, Poland; 2 Institute of Technical Biochemistry, Lodz University of Technology, Stefanowskiego str. 4/10, 90-924, Lodz, Poland; National Renewable Energy Laboratory, UNITED STATES

## Abstract

Samples of bleached kraft pine cellulosic pulp, either treated with an enzyme preparation (a *Thermomyces lanuginosus* xylanase, an *Aspergillus* sp. cellulase, and a multienzyme preparation NS-22086 containing both these activities) or untreated, were refined in a laboratory PFI mill. The treatment with cellulases contained in the last two preparations significantly improved the pulp’s susceptibility to refining (the target freeness value of 30°SR was achieved in a significantly shorter time), increased water retention value (WRV) and fines contents while the weighted average fiber length was significantly reduced. These changes of pulp parameters caused deterioration of paper strength properties. The treatment with the xylanase, which partially hydrolyzed xylan, small amounts of which are associated with cellulose fibers, only slightly loosened the structure of fibers. These subtle changes positively affected the susceptibility of the pulp to refining (refining energy was significantly reduced) and improved the static strength properties of paper. Thus, the treatment of kraft pulps with xylanases may lead to substantial savings of refining energy without negative effects on paper characteristics.

## Introduction

Paper products have been increasingly used for writing and printing as well as for packaging, sanitary-hygiene and special purposes because of the high functionality, relatively low prices, biodegradability and many other attractive properties. Furthermore, paper is produced from renewable biomass feedstocks and waste paper may be either recycled or combusted to generate energy. These advantages cause that production and usage of various sorts of paper have been growing. Only in the first decade of XXI century the global use of paper products increased from 320 milion(mln) tons to 395 milion(mln) tons [1].

Refining of pulps, consisting primarily of cellulose fibers, is a key step of paper making and relies on mechanical treatment of their water suspensions, which increases the fibrillation grade and outer surface area of cellulose fibers that enhances fiber swelling and flexibility as well as bonding interactions between the fibers. The process name was coined several centuries ago and does not precisely reflect its character since it suggests reduction of the cellulose fibers length. Notwithstanding the possible misinterpretation, the term refining is commonly used by pulp and paper technologists and in literature publications [2]. Refining of paper pulps is conducted using either conical or disc refiners in order to loosen the structure of cellulose fibers and this way to produce paper with target properties, meeting end product quality demands. The principal drawback of currently applied refining technologies is the high unit energy consumption, usually ranging from 150 to 500 kWh/ton paper and accounting for 30 to 50% of the total energy used for paper making [3, 4]. For the mean unit energy consumption of 300 kWh per ton and paper production of 400 mln tons/year, the global refining energy reaches 120 TWh, which is equivalent to incineration of around 24 mln tons of black coal per year.

Despite numerous efforts to improve the energy efficiency of conventional disc and conical refiners, this parameter has not been reduced enough yet. The energy efficiency of these refiners ranges from few to over 15% and the majority of refining energy is dissipated as heat, resulting in pulp warming [5, 6]. Since also alternative refining methods (ultrasounds, cavitation, steam explosion, freezing etc.) have not given significant energy savings, chemical (e.g. treatment with bases) and enzymatic methods were proposed to loosen the structure of cellulose fibers before processing in a refiner. Enzymatic treatment of cellulose fibers enables degradation of either all types of associated hemicelluloses or only some of them (e.g. xylans). Removal of these polysaccharides facilitates penetration of water molecules into spaces within cellulose fibers that may break a part of hydrogen bonds connecting cellulose chains. This in turn loosens the 3D structure of fibers, which become more flexible and form paper with more compact structure and high strength [7]. The main objective of application of enzymatic preparations for pulps treatment is to reduce refining energy and make the process not only less expensive but also more environmentally friendly [8]. However, enzymatic treatment must not lead to deterioration of paper properties and therefore selection of suitable enzyme preparations is of key importance. Other criteria of this selection are enzyme’s cost (must not rise the overall process costs) and purity since the presence of additional enzymatic activities (e.g. cellulases apart from xylanases) may negatively affect pulp and paper properties.

In this work, the effect of enzymatic treatment of bleached pine kraft pulp by three different commercial enzyme preparations (a cellulase, a xylanase and a mixture of cellulases and xylanases) on the pulp susceptibility to refining and paper quality was studied. The relationship between the type of glycoside hydrolase (cellulase and/or xylanase) and refining energy consumption required to achieve pulp freeness value of 30°SR as well as most important pulp and paper parameters were evaluated.

## Materials and Methods

### Pulp and its characterization

A bleached pine kraft pulp was kindly donated by one of Polish paper mills. Typically, this pulp is used for production of high quality graphic papers. The pulp had a form of air-dried sheets. The following properties of the unbeaten pulp were determined:

- dry matter content– 96%, the standard deviation of results of four measurements was 0.1%,
- water retention value (WRV)– 93%, (according to the ISO 23714:2014 standard), the standard deviation of results of four measurements was 0.8%,
- weighted average fiber length– 1.76 mm (according to the ISO 16065–2:2014 standard), the measurements were performed in triplicate and the standard deviation was 0.01 mm,
- polymerization degree– 1250 (according to the ISO 5351:2012 standard), the measurements were performed in triplicate and the standard deviation was 40.

### Enzymes and chemicals

A commercial multienzyme (a mixture of cellulases, xylanases and other glycoside hydrolases) preparation NS-22086 was kindly donated by Novozymes A/S (Denmark). Preparations of cellulase from *Aspergillus* sp. and xylanase from *Thermomyces lanuginosus* were purchased from Sigma-Aldrich (USA). All other chemicals were analytical grade and procured from either Sigma-Aldrich (USA) or POCh (Poland).

Activities of cellulases and xylanases were assayed by the 3,5-dinitrosalicylic acid (DNS) method [9] at pH 5.0 and 50°C for 0.5% carboxymethylcellulose (CMC) and 0.5% birch xylan, respectively (reaction time of 5 min). Activities of both the glycosidases were expressed as micromoles of reducing sugars released from the polysaccharide substrates in 1 min (U). The filter paper activity was determined at pH 5.0 and 50°C according to Adney and Baker [10] and expressed in FPU.

### Enzymatic treatments of pulps

Samples of the pulp (22.5 g dry weight) were soaked in water for 24 h before their treatment with enzymes. Then these samples were disintegrated in a laboratory propeller pulp disintegrator (type R1 from Labormeks, Poland) according to the standard ISO 5263–1:2006 and treated with the three listed above enzyme preparations, used separately. After addition of the enzyme preparation to the pulp, the mixture was incubated in a water bath (at 50°C) with shaking (60 rpm) for either 30 minutes (for the *Aspergillus* sp. cellulase and preparation NS 22086) or 60 minutes (for the *T*. *lanuginosus* xylanase). Then the enzymes were inactivated by 10 minute incubation of enzyme-treated pulp in a boiling water bath and the pulp was cooled and filtered through a mesh sieve no. 200. The filtrates were analyzed for glucose concentration using a commercial GOD-POD enzymatic analytical kit (Biomaxima, Poland), to determine the extent of enzymatic pulp degradation.

### Pulp refining and freeness measurements

After inactivation of the enzymes, the pulps were refined using a PFI laboratory mill at 3.4 kG and 1440 rpm, according to the standard ISO 5264–2:2011. The process was accomplished when the pulp freeness value was above 30°SR (its duration was measured with precision of 1 second to calculate the number of mill revolutions). Then, samples of the refined pulp were transferred from the mill to a mixer.

### Pulp quality analysis

The refined pulps were placed in the mixer and the concentration of their suspensions was adjusted to 0.25%. The thoroughly mixed pulp samples were analyzed for the: freeness (according to the standard ISO 5267–1:2002), weighted average fiber length and WRV (according to standards ISO 16065–2:2014 and ISO 23714:2014, respectively) and then were used to produce handsheets of paper.

### Electron microscopy

Changes in the appearance of fibers caused by the action of cellulolytic and xylanolytic enzymes were observed using an electron scanning microscope S-4700 Hitachi SEM/EDS at 200x magnification.

### Paper production and properties

Handsheets of paper were produced with the use of a standard laboratory Rapid-Köthen class sheet former, according to the standard ISO 5269–2:2007.

The following paper properties were measured after conditioning of the handsheets in standard conditions, in compliance with ISO 187:1990 (at air relative humidity φ of 50% and temperature of 23°C):

- grammage—according to ISO 536:2012, only paper sheets of grammage between 74 and 76 g/m2 were accepted for further investigation,
- bulk density—according to PN-EN ISO 534:2012, twelve measurements were made,
- breaking length—according to PN-EN ISO 1924–2:2010, the twelve measurements were made,
- tear resistance—according to PN-EN ISO 1974:2012, the twelve measurements were made.

The complete information related to the experimental data and standard deviation is enclosed in data set available at datadryad.org web page.

## Results

### Enzymatic activities

The applied enzymatic preparations showed activities of both cellulases and xylanases (Table 1). Even the preparation of *T*. *lanuginosus* xylanase displayed the cellulolytic activity for CMC and filter paper. The multienzyme preparation NS-22086 was characterized by the highest activity of cellulases per mass unit (80.58 U/ml for CMC and 112.12 FPU/ml). Its xylanolytic activity was also relatively high (192.48 U/ml for birch xylan). The action of the latter preparation on various cellulosic pulps and lignocellulosic materials was characterized in our former studies [11, 12]. HPLC analyses of hydrolysates produced using this preparation showed that it contains endo-1,4-glucanase, cellobiohydrolase, beta-glucosidase, endo- and exo-type xylanases and other hemicellulases as well as pectinases.

**Table 1:** Activities of cellulases and xylanases (at 50°C and pH 5.0) of the three tested enzyme preparations.

Enzymes	*Aspergillus* sp. cellulase		NS-22086		*T*. *lanuginosus* xylanase	
	U/ml	FPU/ml	U/ml	FPU/ml	U/g	FPU/g
cellulases	2.37	1.96	80.58	112.12	38	122
xylanases	4.68	-	192.48	-	7460	-

The optimal dose of enzymatic preparation and time of pretreatment were selected based on the results of preliminary experiments that are presented in Table 2. The optimal dose of enzyme was selected taking into consideration the maximal tear resistance of paper produced from the pretreated pulp.

**Table 2:** The impact of the dose of enzymatic preparation and pretreatment time on the selected pulp and paper properties.

Enzyme	Dose	Time of pretreatment [min]	Freeness[°SR]	Tear resistance [mN]	Breaking length [m]
*Aspergillus* sp. cellulase (μl/ 1 g s.m.)	45.7	30	16	220	4850
	21	30	16	230	5600
	10.5	30	15	290	6450
	7	30	14	290	6100
NS 22086 (μl/ 1 g s.m.)	4200	5	50	80	3950
	210	5	43	140	4450
	105	15	24	190	5100
	420	30	15	290	5200
	21	30	15	390	5350
	10.5	30	15	370	4900
	4.2	30	14	500	5550
	2.1	60	14	490	5300
*T*. *lanuginosus* xylanase (mg/ 1g s.m.)	10	60	13	800	5150
	20	60	13	770	4950
	2.5	60	14	910	5000

Based on these results, the dose of NS-22086 preparation was set at 2.1 μl/1 g d.w. pulp to minimize the extent of cellulose hydrolysis. This dose corresponded to 0.17 U of cellulase activity for CMC (0.24 FPU) and 0.40 U of xylanase activity per 1 g d.w. pulp. Also doses of the two other enzyme preparations, which were characterized by the higher purity (and price) than the NS-22086 as well as different ratios of cellulase: xylanase activities were relatively low to minimize cellulose hydrolysis.

The doses of the three enzyme preparations added to the pulp were as follows:

- cellulase from *Aspergillus* sp.- 10.5 μl/ 1 g s.m.,
- xylanase from *T*. *lanuginosus–* 2.5 mg/ 1 g s.m.,
- NS 22086–2.1 μl/ 1 g s.m.

Thus the dose of cellulase from *Aspergillus* sp. corresponded to 24.9 x 10^−3^ U of cellulase activity for CMC (20.6 x 10^−3^ FPU) and 49.1 x 10^−3^ U of xylanase activity per 1 g d.w. pulp, which was around 10-fold lower compared to the activities contained in the dose of NS-22086 preparation. The dose of xylanase from *T*. *lanuginosus* corresponded to 0.76 U of cellulase activity for CMC (2.44 FPU) and 149.2 U of xylanase activity per 1 g d.w. pulp. The latter activity was around 47-fold higher compared to the activity of xylanases contained in the dose of NS-22086 preparation while the activities of cellulases were only around 1.8 (for CMC) and 0.8-fold (for filter paper) higher.

One of the products of pulp treatment by the three enzyme preparations was glucose. Its concentrations in the filtrates of enzyme-treated pulp were relatively low, of 0.016, 0.061 and 0.182 mg/ml for the *Aspergillus* sp. cellulase, *T*. *lanuginosus* xylanase and NS-22086 preparation, respectively. Thus even the xylanase preparation released some glucose from the pulp.

### Impact of enzymatic and mechanical treatments on fibre morphology

Pulp freeness is the relatively easily measurable parameter, characterizing refining level and negatively correlated with the pulp dewatering ability, which decreases with the increase in freeness. Because the yield of paper production is diminished when dewatering ability is too low, in industrial conditions freeness values should not be higher than 30°SR.

**Fig 1:**
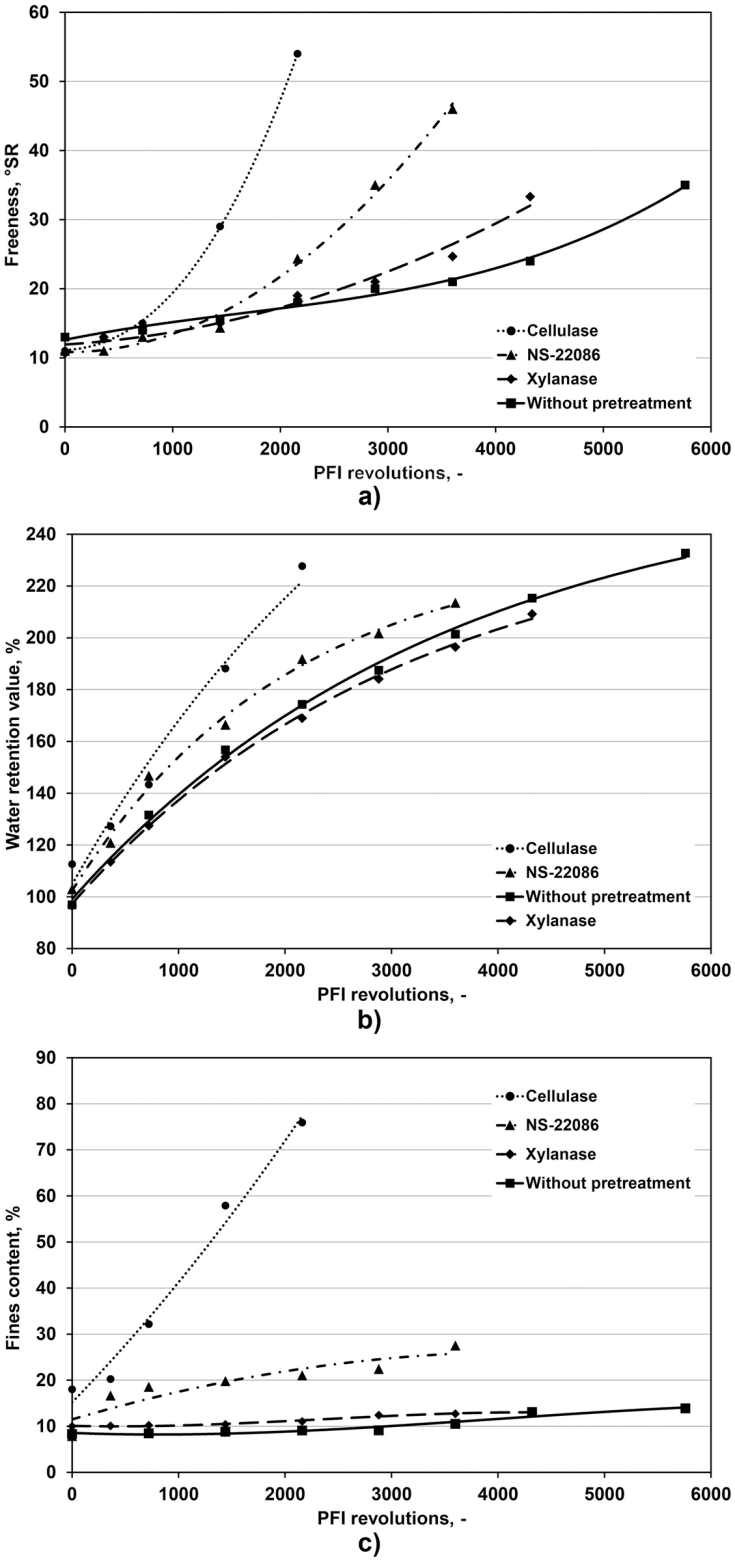
Effects of enzyme treatment and PFI revolutions number on pulp freeness (a), water retention value (b) and fines content (c).

The impact of enzymatic preparations used for pulp treatment on the dynamics of freeness growth during refining is presented in Fig 1A. These results provide evidence that the controlled pulp digestion with the three enzymatic preparations significantly reduced the number of revolutions of the PFI mill, which were necessary to achieve the freeness of 30°SR. This means that the target refining level was achieved in a shorter time. Thus, modification of fiber characteristics by these enzyme preparations significantly reduced refining energy consumption.

The numbers of mill revolutions required to achieve the pulp freeness value of 30°SR are compared in Table 3. The effect of enzymatic pulp treatment with *Aspergillus* sp. cellulase on pulp freeness was stronger than in case of NS-22086 preparation and *T*. *lanuginosus* xylanase (the weakest impact despite the highest cellulolytic and xylanolytic activities used per 1 g d.w. pulp). However, apart from the refined pulp freeness also other parameters such as WRV, average fiber length and fines content decide of paper properties.

**Table 3:** The effect of enzymatic pulp treatment on the number of PFI mill revolutions required to achieve the freeness of 30°SR.

Enzyme preparation	The number of PFI mill revolutions	Percentage of PFI mill revolutions
None	5200	100
cellulase from *Aspergillus* sp.	1500	29
NS-22086	2600	50
xylanase from *T*. *lanuginosus*	4100	79

The data shown in Fig 1B demonstrate that the treatments with the *Aspergillus* sp. cellulase and preparation NS-22086, which contains enzymes hydrolyzing cellulose and xylan, caused that the swelling ability (expressed as WRV) grew significantly faster during refining, compared to the untreated pulp. The influence of *T*. *lanuginosus* xylanase on the growth of this parameter was not so strong. Also the level of fines in the refined pulp was only slightly affected by this enzyme, and interestingly, at the end of pulp refining the percentage of fines was lower the pulp processed with the xylanase than in the untreated pulp (12.8% d.w. versus 13.4% d.w., respectively).

The modification of fibers structure by *Aspergillus* sp. cellulase caused that the percentage of fines was above 70% after around 2000 revolutions of the PFI mill and the resulting refined pulp was useless for paper production. At the freeness of 30°SR the pulp treated with the latter preparation contained as much as 54.5% fines while that treated with NS-22086 preparation contained around twice more fines than the untreated pulp (23.8 versus 13.4% d.w.).

The positive effect of pulp treatment with *Aspergillus* sp. cellulase and NS-22086 preparation before refining on the growth of pulp freeness is in compliance with results reported among others by Cui et al. [13]. Based on experimental results and literature data these authors concluded that the impact of various enzyme preparations on pulp and paper parameters depended on a balance between activities of cellulases and hemicellulases. Our study revealed that pulp treatment with the three enzyme preparations caused the rise in WRV of the pulp during refining and that the impact of *T*. *lanuginosus* xylanase was weaker than the influence of the two cellulases. However, at the end of refining (at the freeness of 30°SR) this parameter was the highest for the untreated pulp (Table 4) while in the study of Liu et al. [14] WRV increased significantly due to the pulp treatment with xylanase before refining. However, also these authors observed the decrease in fines content (in our study their percentage decreased from 13.4 to 12.8%).

**Table 4:** The impact of the three enzyme preparations on refined pulp and paper parameters (for pulp freeness of 30°SR).

Parameter	Enzyme preparation			
	-	*T*. *lanuginosus* xylanase	NS-22086	*Aspergillus* sp. cellulase
PFI mill revolutions number	5200	4100	2600	1500
WRV [%]	225	202	198	194
Average fiber length [mm]	1.66	1.65	1.34	1.04
Fines percentage [%]	13.4	12.8	23.8	54.5
Volumetric mass [g/cm^3^]	0.73	0.70	0.73	0.71
Breaking length [m]	8000	9700	8200	6500
Tear resistance [mN]	500	460	430	210

**Fig 2:**
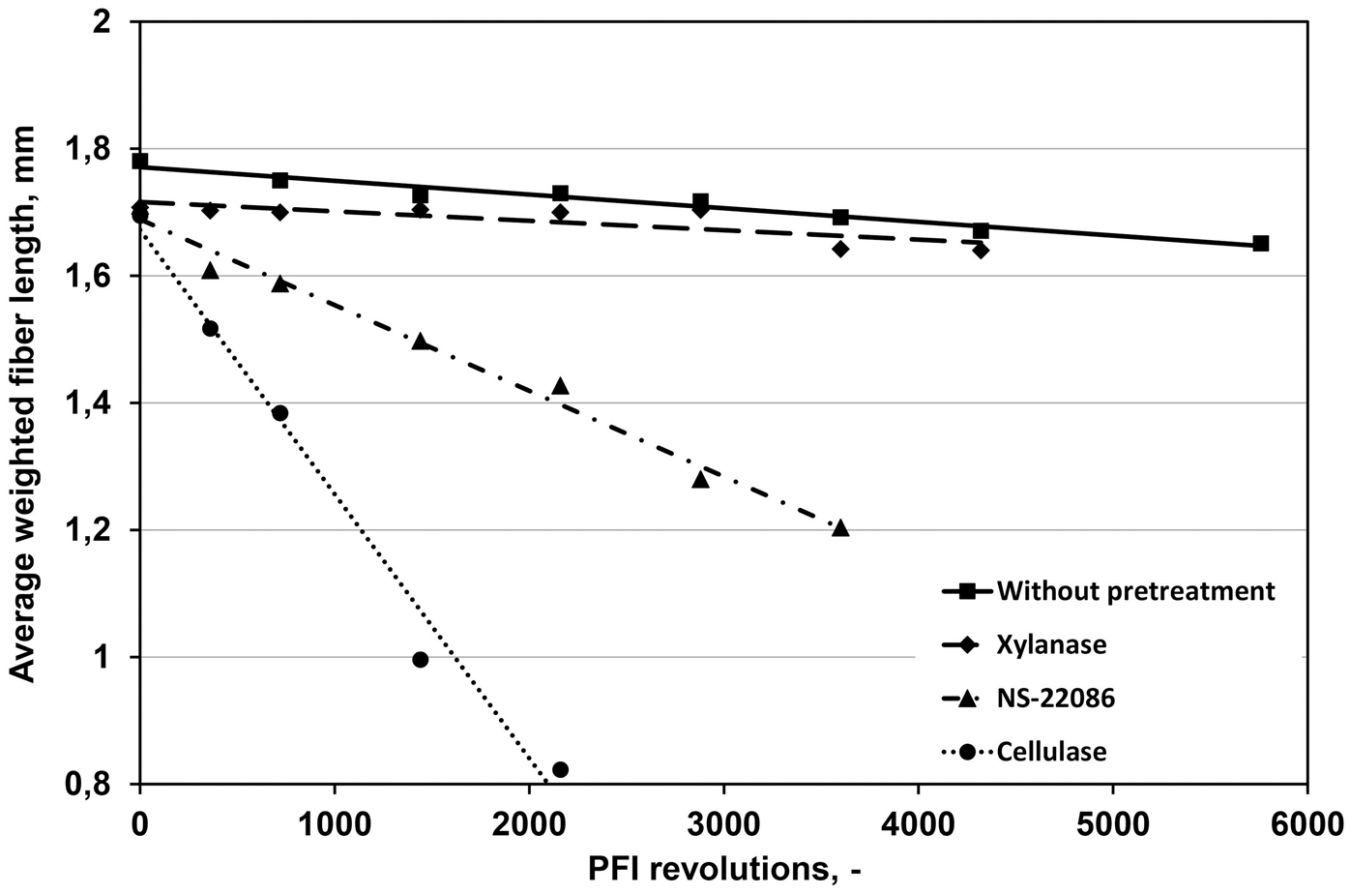
The effect of enzyme preparations and number of PFI mill revolutions on the weighted average fiber length.

Another parameter characterizing the refining outcomes is the weighted average length of fibers, which for the untreated pulp was decreased from 1.76 to 1.66 mm after 5200 revolutions of PFI mill (Fig 2). The decrease in the length of fibers after pulp treatment with the xylanase from *T*. *lanuginosus* was almost the same (from 1.76 to 1.65 mm) while the *Aspergillus* sp. cellulase and multienzyme preparation NS-22086 caused the significant decrease in this parameter (to 1.04 and 1.34 mm at the end of refining, respectively).

These results are consistent with findings of Znidarsic-Plazl et al. [7] who observed that pulp treatment with multienzyme preparations caused the significant decrease in the average fiber length.

### Effect of enzyme treatments on physical properties of handsheets

**Fig 3:**
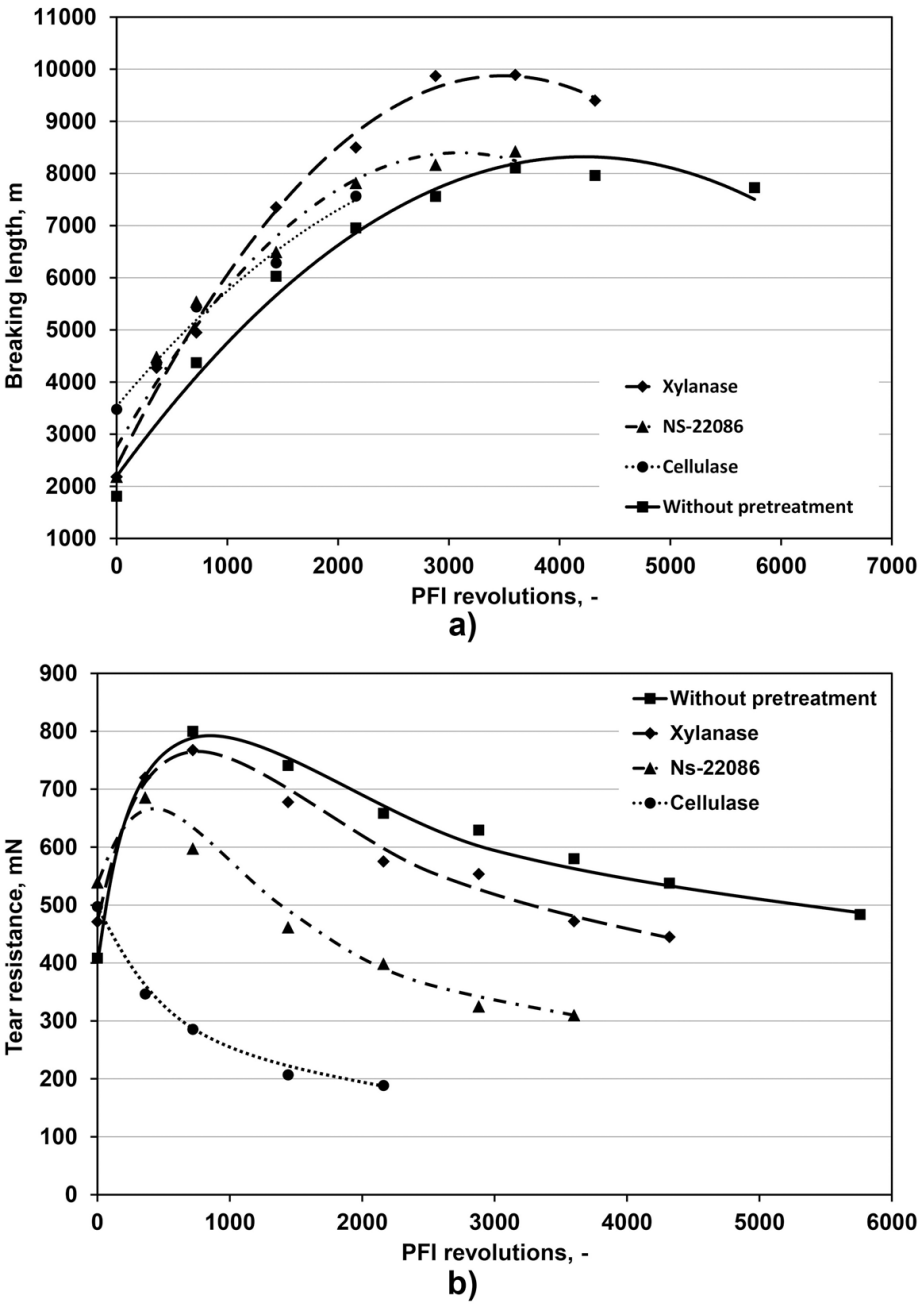
Effects of enzyme treatment and PFI mill revolutions number on breaking length (a) and tear resistance (b) of paper.

The presented above changes in the pulp characteristics caused by partial enzymatic degradation of its components were found to affect properties of paper derived from these pulps. Static paper strength properties are characterized by breaking length. The data presented in Fig 3A demonstrate that pulp treatments with *T*. *lanuginosus* xylanase and NS-22086 preparation increased the maximum value of this parameter from around 8000 m (for the untreated pulp) to around 9700 m and 8200 m, respectively while the treatment with cellulase from *Aspergillus* sp. significantly decreased its value to 6500 m.

The tear resistance of paper characterizes its dynamic strength properties. The results shown in Fig 3B prove that this parameter was reduced by the treatment with each of the three enzymatic preparations (by around 8% for the *T*. *lanuginosus* xylanase, and by around 60% for the *Aspergillus* sp. cellulase and NS-22086 preparation).

The effect of pulp treatment with cellulases on values of breaking length and tear resistance of paper produced from these pulps, observed in our study, is consistent with results reported by Garcia-Ubasart et al. [15] who also observed that the breaking length was positively correlated with the refining level.

**Fig 4:**
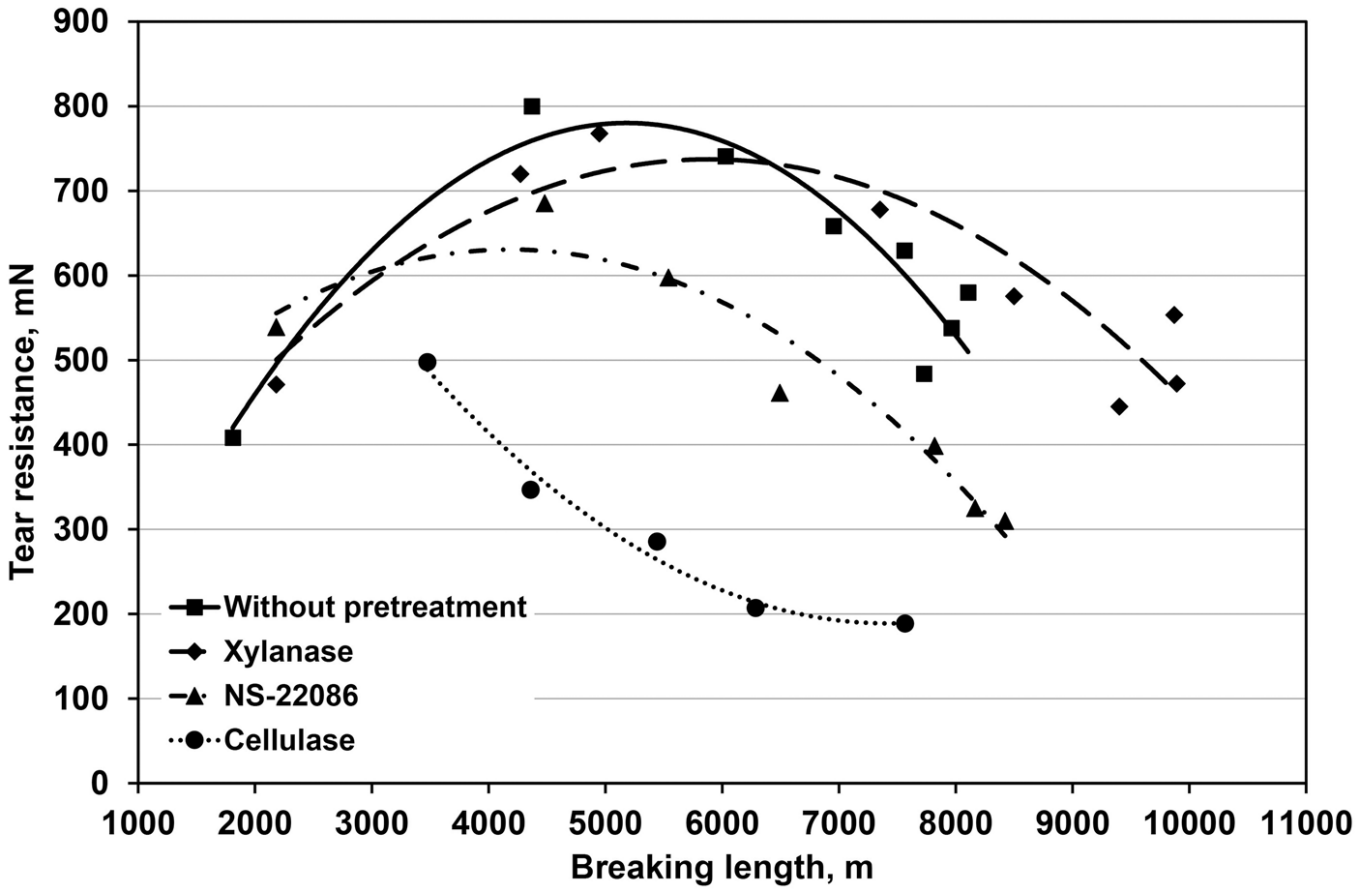
The dependence of tear resistance: breaking length ratio on enzymatic pulp treatment.

The ratio of tear resistance to breaking length is the basic parameter characterizing the strength properties of paper. The data presented in Fig 4 provide evidence that pulp treatment with the *T*. *lanuginosus* xylanase significantly increased this parameter while the two cellulolytic preparations reduced it, which was also reported by Garcia-Ubasart et al. [13].

The impact of the three enzyme preparations on pulp and paper parameters is presented in Table 4 (the refined pulp freeness was 30°SR in each case).

The data collected in Table 4 demonstrate that pulp processing with the tested cellulases and xylanases significantly reduced the time (the number of mill revolutions) during which pulp freeness reached 30°SR, however the action of cellulases on the fibers caused significant deterioration of paper strength properties (reflected by strongly reduced tear resistance and in case of *Aspergillus* sp. cellulase—also breaking length). These properties were not worsened after pulp treatment with the xylanase preparation (despite 8% decrease in tear resistance).

### The impact of pulp enzymatic treatment on refining—microscopic images

**Fig 5:**
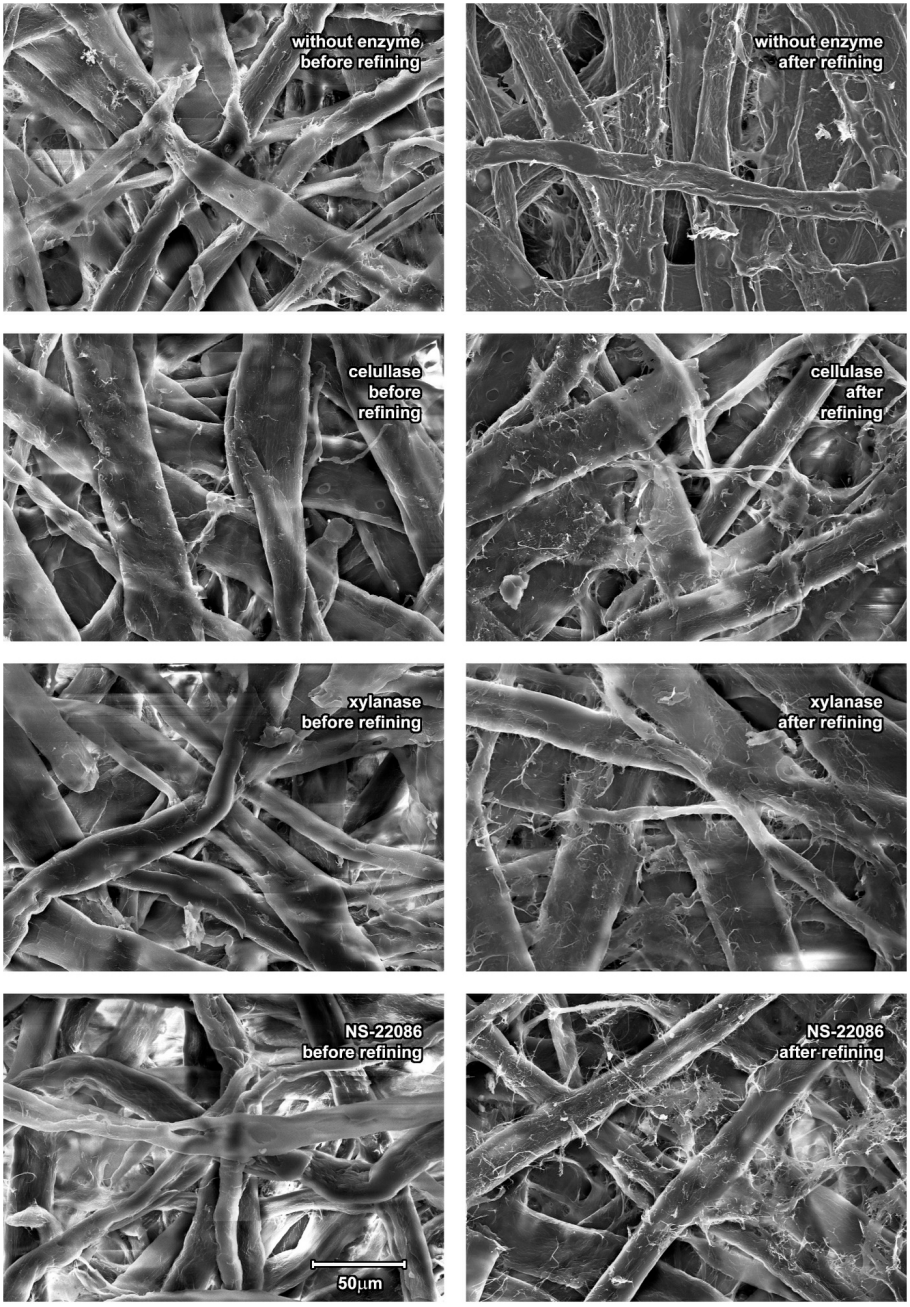
Microscopic images (electron scanning microscope, 50 μm bar enables estimation of the fibres’ size) illustrating the effect of enzymatic pulp treatment on the appearance of fibres before and after refining.

The action of the three enzyme preparations on the pulp was also observed by means of electron microscopy and the microscopic images of enzyme treated and untreated fibers are presented in Fig 5

These images demonstrate that the two preparations of cellulases (from *Aspergillus* sp. and NS-22086) significantly modified the structure of fibers. The damage of cellulose fibers by cellulases was also reported by Garcia-Ubasart et al. [13]. The *T*. *lanuginosus* xylanase only slightly modified the surface of the pulp fibers, which was consistent with images presented by Liu et al. [12].

## Discussion

Enzymatic preparations, particularly those displaying the high cellulolytic and xylanolytic activities, have been increasingly used for bioconversion of lignocellulosic materials to biofuels and numerous valuable chemicals as well as in papermaking. In the latter case, enzymes are used primarily for pulp bleaching, deinking of waste paper and waste water treatment [16]. Enzymes were also found to reduce the pulp refining energy and improve paper strength properties. Studies of many authors focus on enzymatic pulp treatment performed to reduce refining energy and make paper manufacturing more environmentally friendly [17]. In this work, three different preparations such as *T*. *lanuginosus* xylanase, *Aspergillus* sp. cellulase and the multienzyme preparation NS-22086 (containing both cellulases and xylanases) were tested in terms of their influence on pulp and paper properties and potential to reduce energy of pulp refining. The most appropriate of them, in terms of refining energy reduction and maintenance of paper strength properties, was the xylanase preparation.

The effect of xylanases on pulp refining was also studied by Liu et al. [12], Dickson et al. [18] and Batalham et al. [19]. Differences between the published experimental results might be caused by different enzyme: substrate ratios as well as pH and temperature conditions. Our results are to some extent consistent with findings of Liu et al. [12] who observed increased breaking length and tear resistance of paper while our experiments showed that the first parameter was increased by 20% while the second was reduced by 8% due to the pulp treatment with the xylanase. On the contrary, Batalha et al. [17] found that values of both these parameters were decreased in consequence of pulp treatment with xylanase.

Action of the multienzyme preparation NS-22086, showing xylanolytic and cellulolytic activities caused that the time of refining was longer than in case of *Aspergillus* sp. cellulase and shorter than after the treatment with *T*. *lanuginosus* xylanase. The paper strength properties were better after pulp treatment with NS-22086 preparation than after the treatment with cellulase and worse than after the treatment with xylanase. Also Ko et al. [20], Zhang et al. [21], Kim et al. [14] and Lecourt et al. [22] observed that cellulases damaged the fibers, which caused deterioration of paper strength properties, particularly the tear resistance.

Cui et al. [11] postulated that none of industrial enzyme preparations enables both to reduce refining time and maintain or improve paper properties. The same conclusions were presented by Garcia et al. [23], Gil et al. [24], Kim et al. [14] and Oksanen et al. [25].

This study showed that pulps should be treated with preparations characterized by the high xylanolytic activity since the xylanase from *T*. *lanuginosus* reduced the refining time required to reach freeness of 30°SR by around 20% and increased the paper breaking length by around 20%. The only disadvantage caused by the treatment with this enzyme was the 8% decrease in tear resistance.

Unfortunately, the majority of industrial preparations of xylanases contain also cellulolytic enzymes and pure xylanases are relatively expensive. This study provides evidence that the availability of purified xylanases is of key importance for paper making because pulp treatment with these enzymes may give rise to refining energy savings and improved paper strength properties.

## Conclusions

The xylanase from *T*. *lanuginosus* outperformed two preparations of cellulases (*Aspergillus* sp. cellulase and the multienzyme preparation NS-22086, containing also xylanases) when used for kraft pine pulp treatment before refining. All three enzyme preparations significantly reduced the refining energy because the freeness of 30°SR was achieved in a significantly shorter time compared to the untreated pulp. However, partial cellulose degradation by the two preparations of cellulases increased fines contents and reduced the weighted average fiber length that caused deterioration of paper strength properties. The xylanase from *T*. *lanuginosus* partially removed xylan, associated with cellulose fibers, and only slightly loosened their structure and reduced the weighted average fiber length. The removal of xylan not only increased the susceptibility of the pulp to refining but also improved the static strength properties of paper. Thus, the treatment of kraft pulps with robust, cellulase-free preparations of xylanases may not only reduce refining energy but also facilitate production of paper with desirable strength properties.
